# Intralesional Injection of Rose Bengal Induces a Systemic Tumor-Specific Immune Response in Murine Models of Melanoma and Breast Cancer

**DOI:** 10.1371/journal.pone.0068561

**Published:** 2013-07-17

**Authors:** Paul Toomey, Krithika Kodumudi, Amy Weber, Lisa Kuhn, Ellen Moore, Amod A. Sarnaik, Shari Pilon-Thomas

**Affiliations:** 1 H. Lee Moffitt Cancer Center and Research Institute, Immunology Program, Tampa, Florida, United States of America; 2 H. Lee Moffitt Cancer Center and Research Institute, Cutaneous Oncology Program, Tampa, Floria, United States of America; 3 University of South Florida, College of Medicine, Tampa, Florida, United States of America; 4 University of South Florida, Department of Oncologic Sciences, Tampa, Florida, United States of America; Istituto Superiore di Sanità, Italy

## Abstract

Intralesional (IL) injection of PV-10 has shown to induce regression of both injected and non-injected lesions in patients with melanoma. To determine an underlying immune mechanism, the murine B16 melanoma model and the MT-901 breast cancer model were utilized. In BALB/c mice bearing MT-901 breast cancer, injection of PV-10 led to regression of injected and untreated contralateral subcutaneous lesions. In a murine model of melanoma, B16 cells were injected into C57BL/6 mice to establish one subcutaneous tumor and multiple lung lesions. Treatment of the subcutaneous lesion with a single injection of IL PV-10 led to regression of the injected lesion as well as the distant B16 melanoma lung metastases. Anti-tumor immune responses were measured in splenocytes collected from mice treated with IL PBS or PV-10. Splenocytes isolated from tumor bearing mice treated with IL PV-10 demonstrated enhanced tumor-specific IFN-gamma production compared to splenocytes from PBS-treated mice in both models. In addition, a significant increase in lysis of B16 cells by T cells isolated after PV-10 treatment was observed. Transfer of T cells isolated from tumor-bearing mice treated with IL PV-10 led to tumor regression in mice bearing B16 melanoma. These studies establish that IL PV-10 therapy induces tumor-specific T cell-mediated immunity in multiple histologic subtypes and support the concept of combining IL PV10 with immunotherapy for advanced malignancies.

## Introduction

Intralesional (IL) injection of anti-tumor therapeutic agents has been used for the treatment of cutaneous malignancies. IL therapy can control local disease and avoid potential surgical complications and systemic toxicity. While many of these agents lead to regression of injected lesions, systemic responses are rarely observed thus limiting this strategy for patients with metastatic disease. Immunotherapeutic strategies incorporating IL therapy to induce anti-tumor immune responses are currently being explored as a potential means to induce both local and systemic tumor regressions. IL injection of agents that can increase the presentation of tumor-specific antigens, induce infiltration of immune cells, or enhance ongoing immunity may lead to the induction of robust systemic anti-tumor immunity. Intratumoral injection of dendritic cells (DCs) has led to increased lymphocyte infiltration and systemic anti-tumor effects in patients with advanced melanoma [1], [2]. Injection of adjuvants such as BCG or TLR agonists has been shown to enhance tumor-specific immunity [3]–[6]. Intratumoral injection of immune-enhancing cytokines such as IL-2 or GM-CSF has also been shown to enhance anti-tumor immunity in melanoma patients. [6], [7].

Rose Bengal is a water-soluble xanthene dye that had been previously used in liver function studies and is still in use by ophthalmologists [8], [9]. PV-10 is a 10% solution of Rose Bengal formulated for IL injection. IL PV-10 therapy has been shown to induce regression of both injected melanoma lesions and un-injected bystander lesions in patients with melanoma [10]. PV-10 has been documented to induce cell death in melanocytes without affecting normal dermal fibroblasts [11]. Intralesional injection of PV-10 followed by radiotherapy has shown promising results in three patients with metastatic melanoma [12]. Relatively little is known about the mechanism by which PV-10 can induce resolution of bystander lesions. However, an immune-mediated process is likely as responses in untreated lesions occur only when there is response in injected lesions, and regression of bystander lesions typically occur in a delayed fashion compared to regression of injected lesions. This study was undertaken to elucidate the apparent immune mechanism of this systemic effect following lesion ablation with PV-10.

## Materials and Methods

### Animals

This study was carried out in strict accordance with the recommendations in the Guide for the Care and Use of Laboratory Animals of the National Institutes of Health. The protocol was reviewed and approved by the Institutional Animal Care and Use Committee at the University of South Florida (#A4100-01). Mice were humanely euthanized by CO_2_ inhalation according to the American Veterinary Medical Association Guidelines. Mice were observed daily and humanely euthanized if a solitary subcutaneous tumor exceeded 1.5 cm in diameter or mice showed signs referable to metastatic cancer. For mice bearing lung metastases, mice were humanely euthanatized at day 21 after intravenous (i.v.) injection of tumor and tissues were harvested. All efforts were made to minimize suffering. Female C57BL/6 or BALB/c mice (6–8 weeks old) were purchased from Harlan Laboratories (Indianapolis IN). Mice were housed at the Animal Research Facility of the H. Lee Moffitt Cancer Center and Research Institute.

### Cell Culture and PV-10

The B16 melanoma cell line is a tumor of spontaneous origin in C57BL/6 mice. The MT-901 breast adenocarcinoma is of BALB/c origin. The MC-38 (C57BL/6 origin) and CT-26 (BALB/c origin) cell lines were utilized as irrelevant controls. Cell lines were cultured in T-150 flasks in complete media (CM), which consisted of RPMI supplemented with 10% of heat inactivated fetal bovine serum, 0.1 mM nonessential amino acids, 1 µM sodium pyruvate, 2 mM fresh L-glutamine, 100 µg/ml streptomycin, 100 units/ml penicillin, 50 µg/ml gentamicin, 0.5 µg/ml fungizone (all from Life Technologies, Inc. Rockville, MD) and 0.05 mM 2-mercaptoethanol (Sigma Chemical Co., St. Louis, MO.). Single cell suspensions of tumor cells were prepared for injection by washing extensively with PBS. PV-10 (provided by Provectus Pharmaceuticals Inc., Knoxville, TN) is a sterile, non-pyrogenic 10% solution of Rose Bengal disodium in 0.9% saline.

### Induction of Bilateral Flank Subcutaneous MT-901 Lesions

BALB/c mice were injected in the bilateral flanks s.c. with 1×10^5^ MT-901 cells. Seven days later, mice were treated with 50 µL of PV-10 injected intralesionally into the tumor on the right flank. Control mice received intralesional injection with 50 µl of PBS. Tumor sizes were measured for both the right (treated) and left (untreated/bystander) tumors.

### Induction of Lung and Subcutaneous B16 Lesions

C57BL/6 mice were injected via the intravenous route with 5×10^5^ viable B16–F10 and subcutaneously (s.c.) with 1×10^5^ B16 cells. Seven days later, mice were treated by intralesional injection of the subcutaneous tumor with 50 µl PV-10. Control mice received intralesional injection with 50 µl of PBS. Lungs were harvested on day 21. Pulmonary lesions were enumerated after insufflation of the lungs with Feketes solution (100 mL of 70% alcohol, 10 mL formalin, and 4 mL glacial acetic acid).

### Isolation of Splenocytes

Spleens from PV-10 treated or control mice were prepared as single-cell suspensions followed by lysis of red blood cells with ACK lysis buffer, washed twice with PBS, counted and used for *in vitro* assays.

### Flow Cytometry (FACS)

Splenocytes were stained with the following monoclonal antibodies for flow cytometric analysis: anti-mouse CD3, CD8, CD4, CD25, FoxP3, CD56, CD19, CD11b, Gr-1, and F4/80. For the intracellular staining of FoxP3, cells underwent surface staining and then were permeabilized and fixed using an intracellular stain kit (BD Biosciences).

### Interferon-γ ELISA

Splenocytes isolated from C57BL/6 mice were plated in CM alone or at a 10∶1 ratio with irradiated B16 or MC-38 cells (irrelevant control). Splenocytes isolated from BALB/c mice were plated in CM alone or at a 10∶1 ratio with irradiated MT-901 or CT-26 cells (irrelevant control). Supernatants were obtained after 24 and 48 hours. The IFN-gamma concentration in supernatants was evaluated using OptEIA murine ELISA sets (BD Biosciences, San Jose, CA).

### Cytotoxic Assay

A ^51^Cr release assay was done as described previously [13]. Briefly, spleens were harvested from PBS or PV-10 treated mice on day 21. T cells were purified using T cell columns (R& D systems) and were restimulated with irradiated B16 tumor cells for seven days and used as effector cells. Target B16 and MC-38 cells were labeled with 100 µCi of ^51^Cr (Perkin Elmer, Waltham, MA) at 37°C in a 5% CO_2_ atmosphere for one and half hours. The labeled tumor cells were washed three times and added to the effector cells in triplicate wells of 96-well round-bottomed microplates at 200∶1, 100∶1, and 50∶1 effector to target ratios. After 5 hours, the percentage of specific ^51^Cr release was determined by the following equation: [(experimental cpm − spontaneous cpm)/(total cpm incorporated − spontaneous cpm)] × 100. All determinations were done in triplicate, and the SE of all assays was calculated and was typically 5% of the mean or less.

### Adoptive T Cell Therapy

A total of 1×10^5^ B16 tumor cells were injected s.c. in the left flank of C57BL/6 mice. Three days later, mice received a sublethal dose (600 cGy) of total body irradiation (TBI) administered by a [^137^Cs] γ radiation source. For T cell transfer, spleens were harvested from PV-10 treated mice that rejected tumor and T cells were purified using T cell columns (R& D systems). Mice received 1×10^7^ T cells/mouse i.v. on day four after tumor injection. The control group received PBS alone. Tumor size was measured and recorded every two days.

### Statistical Analysis

A Mann-Whitney U test was utilized for comparison of lung lesions and tumor sizes across time points. For *in vitro* assays, an unpaired t-test was utilized to compare between two treatment groups. All statistical evaluations of data were performed using GraphPad Prism software (GraphPad, La Jolla, CA). Statistical significance was achieved at p<0.05.

## Results

### Efficacy of PV-10 in the MT-901 Breast Cancer Model

**Figure 1:**
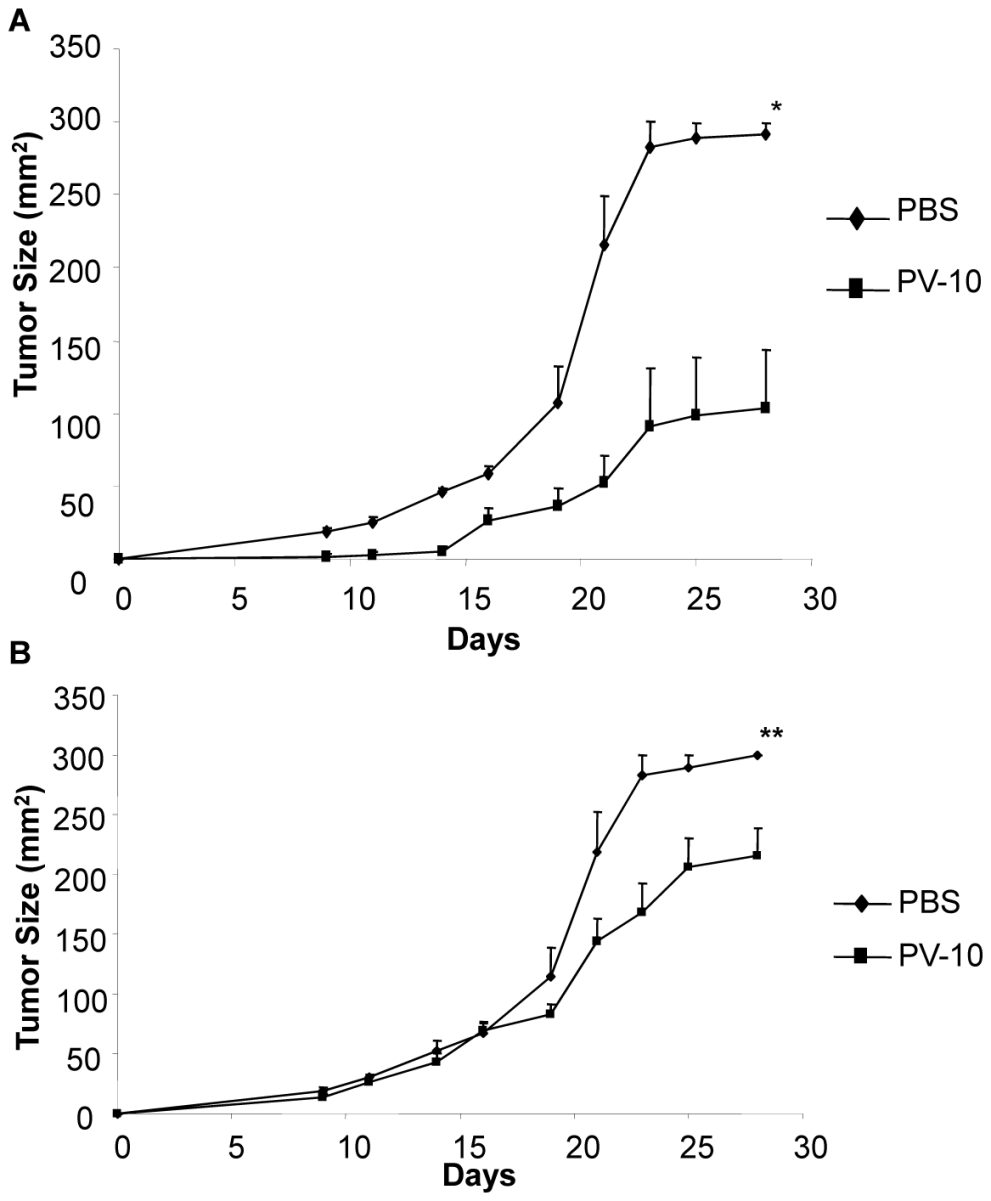
Treatment with PV-10 leads to tumor regression in MT-901 breast cancer model. BALB/c mice (n = 8 mice per group) were injected on each flank with MT-901 cells. Seven days later, mice received intratumoral injection of the right flank lesion with 50 µl PV-10. Tumor growth was measured in (A) treated and (B) untreated tumors. Data shows the mean ± SEM for each time point. Experiment was repeated two times with similar results. *indicates p<0.001, **indicates p<0.05.

BALB/c mice were injected on each flank with MT-901 cells. Seven days later, mice received IL injection of the right flank lesion with 50 µl PV-10. As shown in Figure 1A, significantly smaller tumors were observed in mice treated with PV-10 (p<0.001 compared to PBS-treated mice). In addition, smaller tumor sizes were measured in the bilateral (untreated) tumors of mice treated with PV-10 (Figure 1B, p<0.05 compared to PBS treated mice).

**Figure 2:**
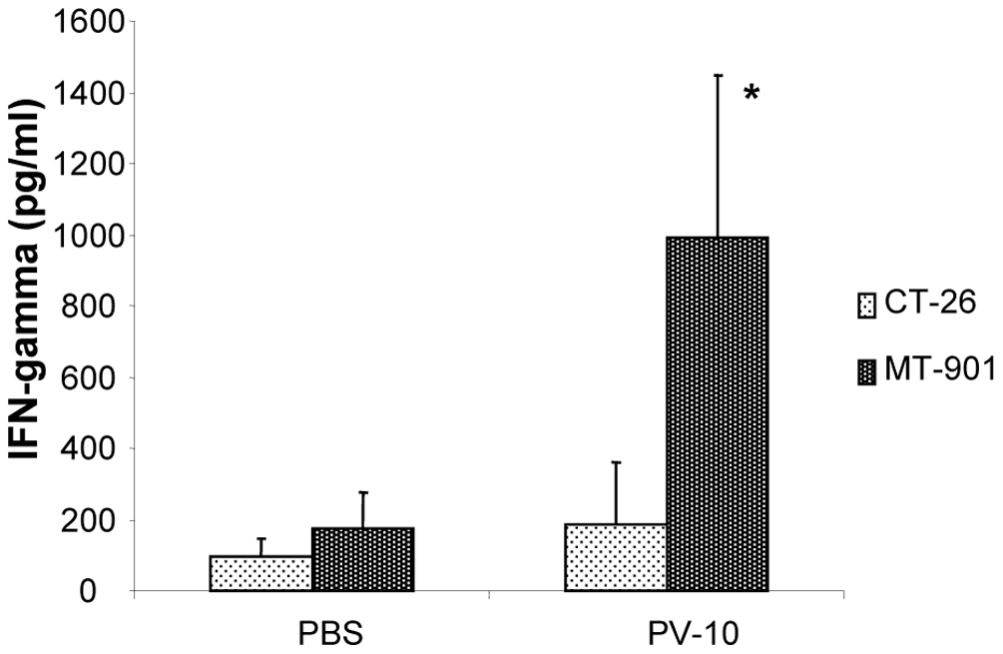
PV-10 treatment leads to tumor-specific IFN-gamma responses in mice bearing MT-901 breast cancer. Splenocytes were plated at 2×10^6^, co-cultured with 2×10^5^ irradiated MT-901 tumor cells, and incubated for 48 hours. Culture supernatants were analyzed for IFN-γ production using commercially available ELISA kit. Data shows the mean ± SD of triplicates. *indicates p<0.05.

We next examined induction of tumor-specific immunity in MT901-bearing mice. Mice were injected s.c. with MT901 cells on the flank. On day 7, mice received an intralesional injection of PBS or PV-10. Spleens were collected on day 21 after treatment. Splenocytes were re-stimulated with MT-901 cells for 48 hours, and supernatants were collected. As shown in Figure 2, mice that received PV-10 produced increased IFN-γ in response to MT-901 cells compared to PBS-treated mice (p<0.05). No IFN-γ production was measured in response to irrelevant control CT-26 cells.

### Efficacy of PV-10 in the B16 Melanoma Model

**Figure 3:**
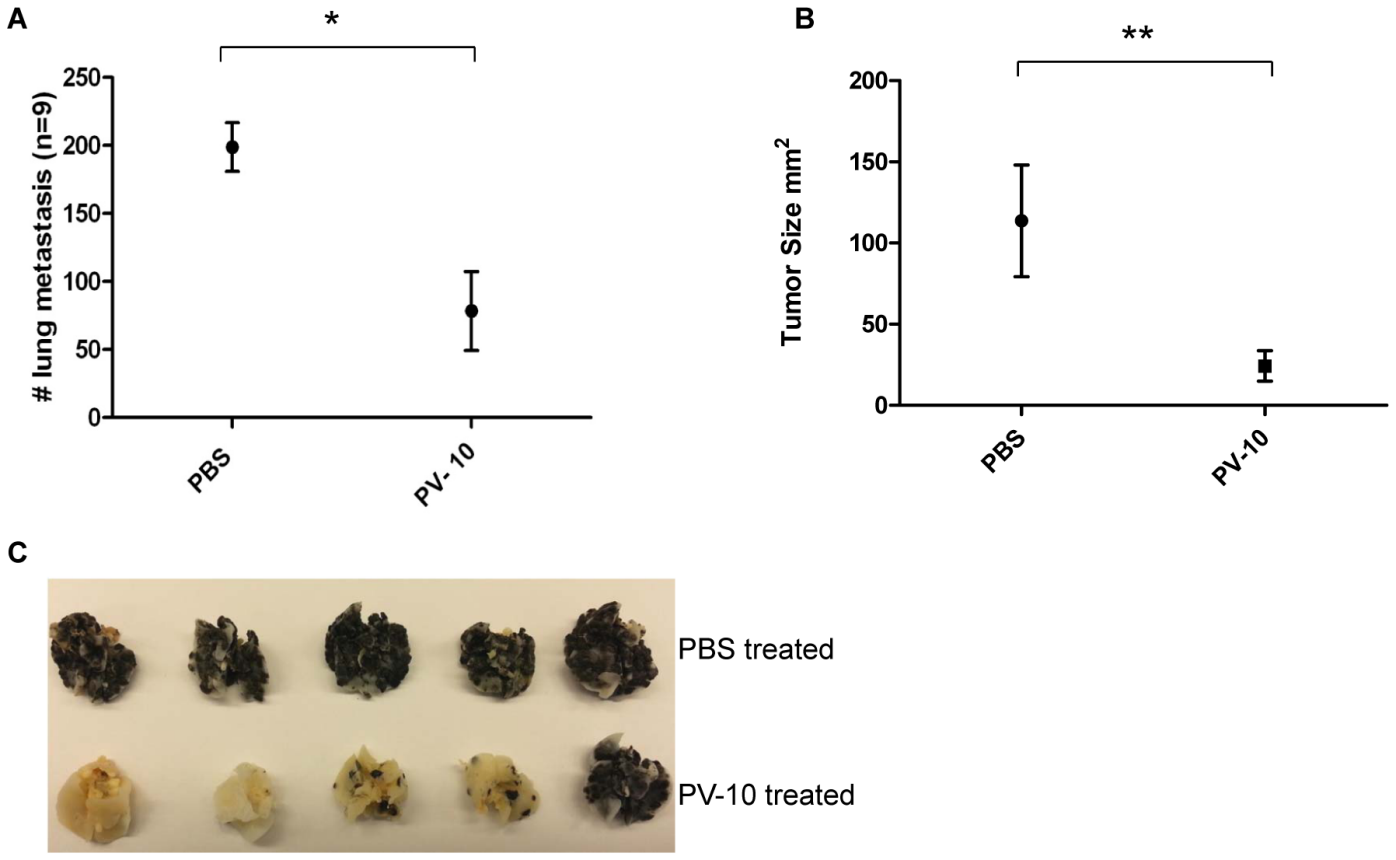
IL injection of PV-10 demonstrates systemic effect on the regression of B16 lung metastases. C57BL/6 mice (n = 10 per group) were injected intravenously with 5×10^5^ viable B16 cells and subcutaneously (s.c.) with 1×10^5^ B16 cells. Seven days later, mice were treated by intralesional injection of the subcutaneous tumor with 50 µl PV-10. Lungs were harvested on day 21. Pulmonary lesions were enumerated after insufflation of the lungs with Feketes solution. (A) Dots represent the mean number ± SD of lung lesions. (B) Dots represent the mean size ± SD of subcutaneous tumors. Experiment repeated three times with similar results. *indicates p<0.01; **indicates p<0.05. (C) Representative lungs from treated mice.

In a more aggressive model, mice received 1×10^5^ B16 cells s.c. on day 0 to establish a solitary tumor on the flank and 5×10^5^ B16 cells intravenously (i.v.) to establish multiple lung lesions. On day 7, mice were treated with 50 µl PBS or PV-10 IL to the flank tumor. Growth of s.c. tumors was measured until day 21 when mice were sacrificed to enumerate lung lesions. All mice that received PBS treatments displayed growth of tumor in the flank and had more than 250 lung lesions. In contrast, mice that received IL PV-10 demonstrated fewer lung lesions (Figure 3A, p<0.01 compared to PBS-treated mice) and had significantly smaller subcutaneous tumors (Figure 3B, p<0.05 compared to mice treated with IL PBS). Representative lungs are shown in Figure 3C. To determine whether injection of the s.c. B16 tumor was required for the observed regression of tumor in the lungs, mice bearing B16 lung lesions were injected s.c. with 50 µl of PV-10 into a non-tumor bearing flank. No difference was measured in the number of B16 lung lesions in mice treated s.c. with PBS or PV-10 (not shown). This indicates that direct injection of PV-10 into a tumor lesion is required for the observed systemic effect of PV-10.

**Figure 4:**
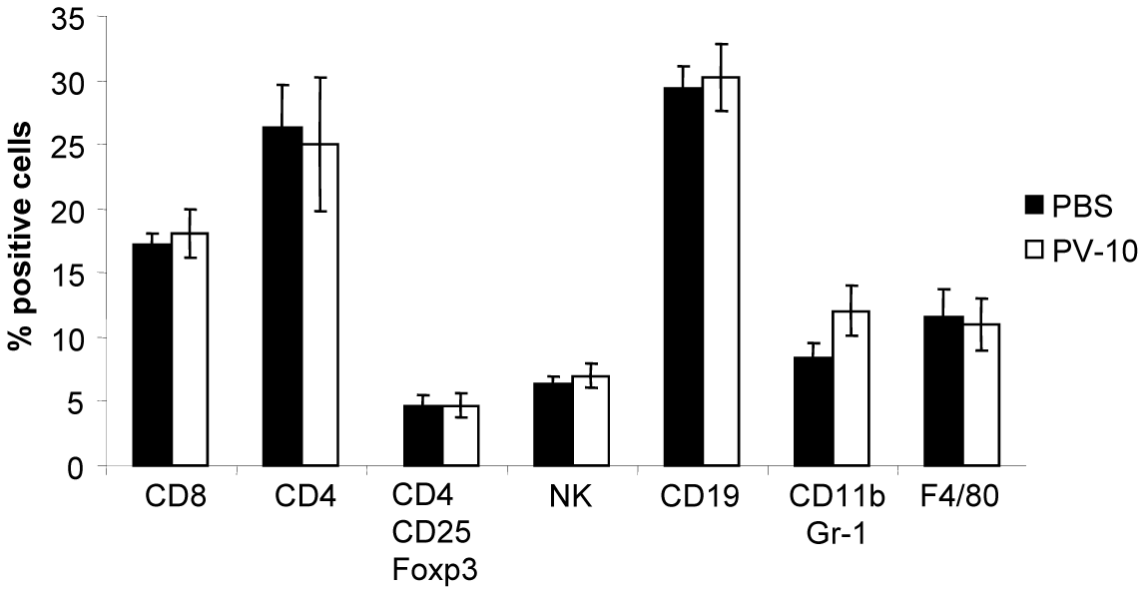
Effect of PV-10 on immune subsets. B16 tumor bearing mice were treated on day 7 with a single IL injection of 50 mcL PBS or PV-10. Spleens were collected 7 days after injection and stained with specific antibodies for flow cytometric analysis.

To determine whether PV-10 has a direct effect on the frequency of immune cell subsets, mice bearing B16 tumor in the flank were treated on day 7 with a single IL injection of 50 µL PBS or PV-10. Spleens were collected 7 days after injection. As shown in Figure 4, no differences were measured in the percentage of T cells (CD8^+^ or CD4^+^), regulatory T cells (CD4^+^ CD25^+^ Foxp3^+^), NK cells, B cells (CD19^+^), myeloid derived suppressor cells (CD11b^+^Gr1^+^) or macrophages (F4/80^+^) in splenocytes of PV-10 treated mice. No differences were observed in cell subsets on day 21 (not shown).

**Figure 5:**
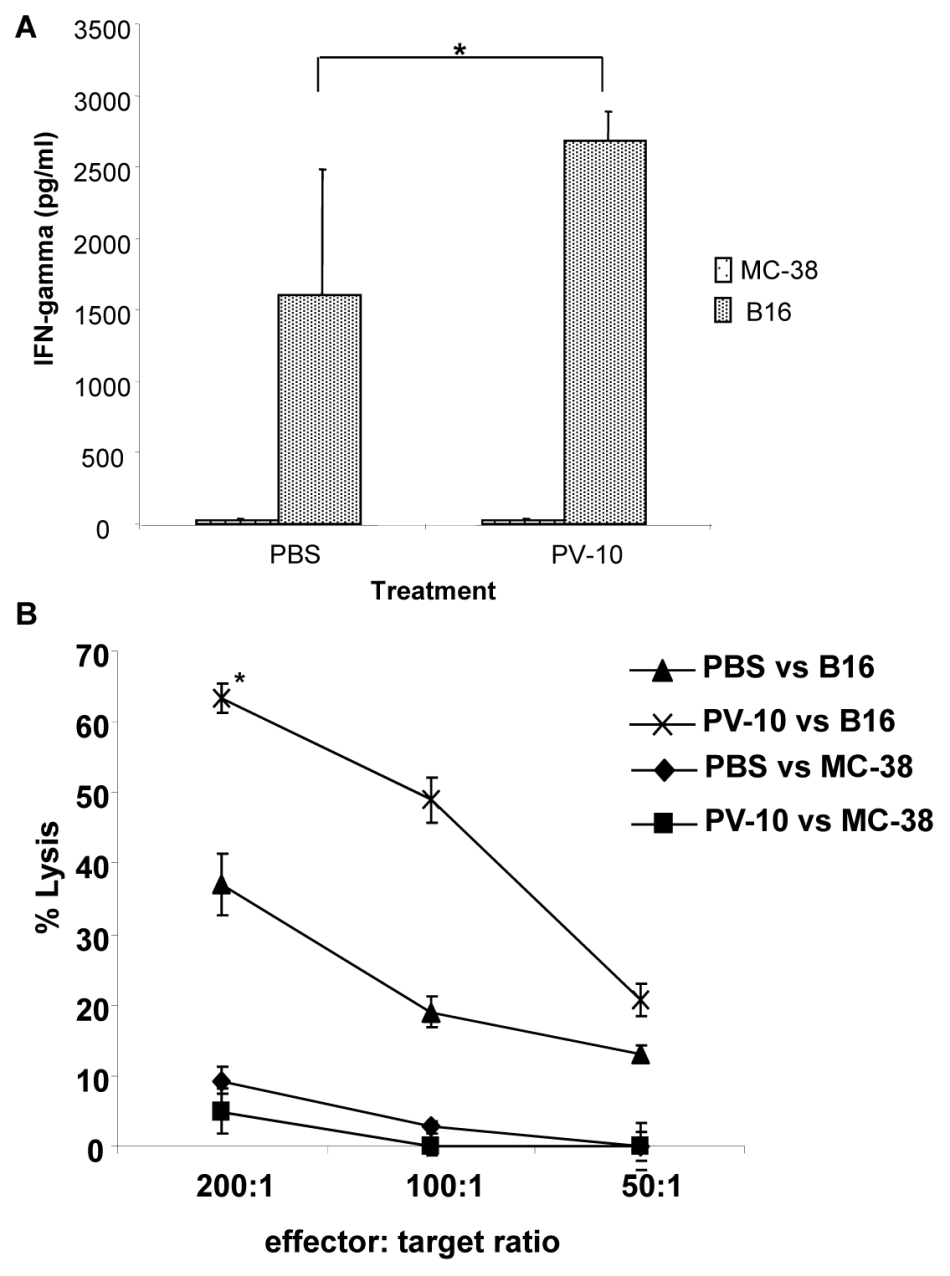
PV-10 treatment leads to tumor specific T cell response in B16 melanoma model. (A) Splenocytes were plated at 2×10^6^, co-cultured with 2×10^5^ irradiated B16 or MC-38 tumor cells, and incubated for 48 hours. Culture supernatants were analyzed for IFN-γ production using commercially available ELISA kit. Data shows the mean ± SD of triplicates. (B) A five hour ^51^Cr release assay was performed using B16 or MC-38 tumor cells as targets. T cells purified from spleens of PBS or PV-10 treated mice were used as effector cells. Data shows the mean ± SE of triplicates from a chromium release assay. *indicates p<0.001.

To determine whether B16-specific T cells were induced after injection with PV-10, mice bearing B16 tumor were treated on day 7 with a single IL injection of 50 µL PBS or PV-10. One week later, spleens were collected and splenocytes were restimulated with B16 or MC-38 cells. IFN-γ was measured in the supernatants after 48 hours. Splenocytes isolated from B16-bearing mice treated with PV-10 demonstrated a significant increase in production of IFN-γ compared to PBS-treated mice (Figure 5A). No IFN-γ production was demonstrated in response to irrelevant control MC-38 cells in either treatment group. Flow cytometric analysis demonstrated that CD8^+^ T cells were producing IFN-γ (not shown).

Next, we measured the ability of T cells in splenocytes to lyse B16 cells. As shown in Figure 5B, T cells from PV-10 treated mice demonstrated a significant increase in cytolytic activity against B16 cells compared to T cells from PBS treated mice. T cells did not lyse irrelevant control MC-38 cells. These studies demonstrate the induction of melanoma-specific immunity in mice treated with PV-10.

### Adoptive Transfer of T cells

**Figure 6:**
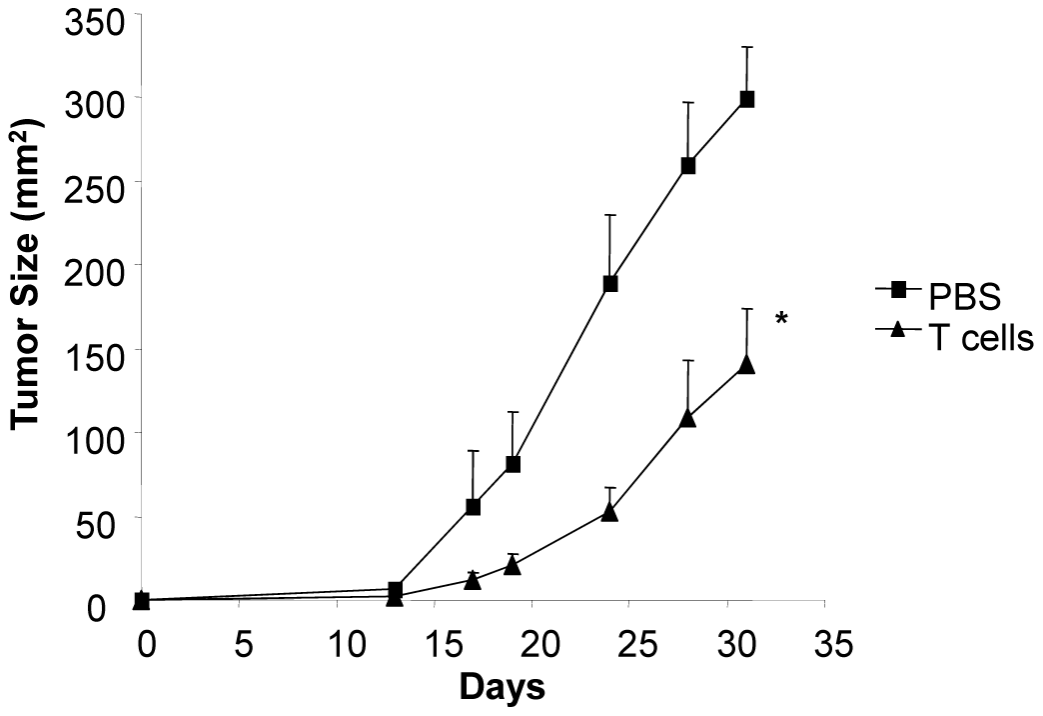
Adoptive transfer of T cells from PV-10 treated mice leads to delay in tumor growth in B16 tumor bearing mice. A total of 1×10^5^ B16 cells were injected s.c. in the left flank of C57BL/6 mice. Three days later, mice received a sublethal dose (600 cGy) of total body irradiation (TBI). For T cell transfer, mice received 1×10^7^ T cells/mouse on day 4. Mice in the control group received only PBS. *indicates p<0.01.

We next determined if anti-B16 immunity of T cells from PV-10 treated mice could be adoptively transferred. Mice bearing B16 tumor were treated on day 7 with a single IL injection of 50 µL PV-10. T cells were purified from the spleens of mice that rejected B16 tumor challenge after 3 weeks of PV-10 treatment. Recipient mice were injected with B16 cells and treated with 600 cGy of total body irradiation on day 3 after tumor injection. One day later, mice received an i.v. injection with purified T cells. Control mice received PBS. As shown in Figure 6, a significant delay in tumor growth was measured in mice receiving T cells (p<0.01 compared to mice treated with PBS).

## Discussion

Intralesional (IL) therapies for melanoma have been undertaken for over forty years with a variety of agents including cytokines, pathogens and various pharmaceutical drugs. Some of these agents, such as intralesional IL-2 therapy, were found to be effective for inducing local disease control [14]. However, few intralesional agents have induced a systemic response evidenced by the regression of bystander, untreated lesions. Bacillus Calmette-Guérin (BCG) is the most extensively studied of these agents. BCG has been used since the 1970s for local control of melanoma and is able to induce regression of untreated lesions in 15–20% of patients [15]–[19].

Recently, PV-10 has been used as an IL therapy for malignancies including melanoma. In initial clinical testing, PV-10 therapy induced regression of both uninjected as well as injected melanoma lesions [10]. Intralesional BCG has been associated with patient fatalities due to anaphylactic hypersensitivity reactions that have not been reported with PV-10 [20]–[24]. In mice, it has been shown that repeated injections of high dose BCG by the s.c route led to mortality [25], indicating that PV-10 may be safer than BCG for intralesional therapy. In this study, we verified that IL PV-10 resulted in regression of untreated bystander lesions in breast cancer and melanoma mouse models. In both models, IL PV-10 was associated with enhanced tumor-specific interferon-γ secretion. These results confirm that IL PV-10 can induce a systemic anti-tumor immune response that can mediate the regression of untreated lesions.

The mechanism by which PV-10 induces systemic immunity is currently unknown. Our studies have shown that direct injection into a tumor lesion is required for the systemic effects of PV-10 as injection of PV-10 into the flank had no effect on distant lung lesions (not shown). After IL injection, PV-10 has been shown to accumulate in the lysosomes of tumor cells resulting in tumor necrosis [11]. It is possible that tumor ablation by PV-10 leads to the release of large amounts of tumor debris that is taken up by antigen-presenting cells such as DCs. The effect of PV-10 on DCs and immune cell infiltration into treated tumor lesions is currently being explored.

While IL-PV-10 therapy alone is capable of inducing an effective systemic anti-tumor immune response, combination of IL PV-10 with other forms of immunotherapy may lead to enhanced responses. It has been shown that tumor ablation in combination with anti-CTLA-4 antibody leads to enhanced anti-tumor immunity in the B16 model [26]. We have shown that direct intratumoral injection with CpG can enhance anti-tumor effects mediated by DC vaccinations [5]. In our study, we demonstrated that systemic anti-tumor immunity in untreated tumor-bearing mice could be mediated by the adoptive transfer of T cells isolated from PV-10 treated mice. As our group has extensive experience with adoptive T cell therapy for the treatment of metastatic melanoma [27], [28], our results support the hypothesis that PV-10 treatment may be combined with adoptive cell therapy to boost the immune response in patients subsequently undergoing adoptive cell therapy.

These studies have demonstrated that intralesional PV-10, in addition to reducing the growth of a directly injected tumor, leads to the induction of a robust anti-tumor T cell response and supports the use of PV-10 to induce systemic anti-tumor immunity for the treatment of metastatic melanoma and breast cancer.
